# How does urbanization affect perceptions and traditional knowledge of medicinal plants?

**DOI:** 10.1186/s13002-021-00473-w

**Published:** 2021-08-03

**Authors:** Cecilia Arjona-García, José Blancas, Leonardo Beltrán-Rodríguez, Citlalli López Binnqüist, Hortensia Colín Bahena, Ana Isabel Moreno-Calles, José Antonio Sierra-Huelsz, Xavier López-Medellín

**Affiliations:** 1 Centro de Investigación en Biodiversidad y Conservación (CIByC), Universidad Autónoma del Estado de Morelos, Av. Universidad 1001, Colonia Chamilpa, C.P, 62209 Cuernavaca, Morelos Mexico; 2 Jardín Botánico, Instituto de Biología, Universidad Nacional Autónoma de México, Tercer Circuito exterior, S/N Ciudad Universitaria, Coyoacán, C.P, 04510 Mexico City, Mexico; 3 Centro de Investigaciones Tropicales (CITRO), Universidad Veracruzana, José María Morelos 44, Zona Centro, Centro, C.P. 91000, Xalapa-Enríquez, Veracruz, Mexico; 4 Centro de Investigaciones Biológicas (CIB), Universidad Autónoma del Estado de Morelos, Av. Universidad 1001, Colonia Chamilpa, C.P, 62209 Cuernavaca, Morelos Mexico; 5 Escuela de Estudios Superiores – Campus Morelia, Universidad Nacional Autónoma de México, Antigua Carretera a Pátzcuaro 8701, Residencial San José de la Huerta, C.P, 58190 Morelia, Michoacán Mexico; 6 People and Plants International, Bristol, VT 05443 USA

**Keywords:** Biocultural conservation, Cultural changes, Ethnobotany, Traditional knowledge, Tropical deciduous forest

## Abstract

**Background:**

The use and knowledge of medicinal plants play an essential role in community health in rural Mexico. Medicinal plants are part of the local heritage and provide a source of economic income. Nevertheless, knowledge of their use has declined due to factors like accelerated urbanization. Some authors have proposed that by reducing natural spaces, urbanization generates changes that impact the recognition, use, and management of natural resources. Here, we evaluate how urbanization affects the knowledge, use, and perception of medicinal plants in a Biosphere Reserve in Mexico.

**Methods:**

Using a mixed methodology including quantitative and qualitative analyses, we generated a list of medicinal plants, methods of preparation, prevalence of illness, and use in two communities with different degrees of urbanization.

**Results:**

A total of 217 medicinal plants were identified. The more urbanized community had greater knowledge of, and used, a larger number of introduced plant species, while the less urbanized community used and had more knowledge about wild plants. One of the factors explaining these differences was occupation, with people who work outdoors showing greater knowledge of wild plants.

**Conclusions:**

Urbanization can lead to a loss of knowledge of the use and management of local wild species, with implications for the conservation of biocultural heritage. Substitution of native medicinal plants by introduced species shows disinterest and disuse in the local medicinal flora, which could be reflected in their ecosystems.

## Background

Traditional knowledge of the use and management of natural resources is a reflection of the relationship between human communities and their physical, biotic, and cultural environment over time [1, 2]. This relationship is mediated by the cultural, economic, and ecological context, making it dynamic and versatile [3, 4]. These changes can modify traditional knowledge, such that it grows, remains the same, or erodes [3]. This can affect how elements of nature are used and managed, as well as practices, customs, beliefs, and ideas [5, 6] at both the individual and group levels [4]. Consequently, there is a consensus that biodiversity conservation implicitly involves traditional knowledge [7, 8].

Some studies have shown that processes associated with modernization negatively affect the degree and depth of knowledge of natural resources; increasing educational level, migration, and urbanization are related to loss of the ability to recognize, name, use, and manage plant resources [9–11]. Urbanization is a complex economic process that entails social and environmental changes that occur over short time periods and often modify cultural patterns [6, 12]. This process sometimes generates innovations in the culture that, in association with the acquisition of prestige, motivate the displacement of patterns of social behavior and organization [13]. At the same time, urbanization leads to drastic changes in people’s lifestyles, perceptions, and sociability [14, 15], which can directly affect the use and management of natural resources.

Urbanization transforms land use and radically changes ecological patterns and processes [16]. Urbanization often includes the removal and logging of large areas of forests to make way for human settlements of various kinds [17]. Thus, urbanization results in a profound transformation of the environment, generating alterations in biogeochemical cycles, habitat fragmentation, and changes in the abundance, diversity, and composition of species [16, 18, 19]. It also generates changes in the ways of feeding and in the vocation of agroecosystems [20–22]. At a cognitive level, urbanization can lead to a disconnect between people and the natural environment, causing what Pyle (1993) [23] calls “the extinction of the experience.”

In particular, urbanization can affect people’s knowledge of medicinal plants, which includes recognizing, naming, using, and managing species in that use category. It has been hypothesized that urban communities, by having increased access to medical services, may abandon or reduce their use of medicinal plants to treat some illnesses and ailments [24, 25]. In addition, this loss of knowledge and abandonment of use could be due to a decrease in agricultural, agroforestry, and forested areas, since urbanization reduces the areas for medicinal plant collection. At the same time, urbanization decreases people’s involvement in activities in natural environments and can lead to devaluation of and discrimination against traditional knowledge. Some authors have suggested that the decreased contact between people and their natural environment results in societies that are more tolerant of the progressive loss of biodiversity [26]. Therefore, the management and transmission of traditional knowledge to new generations is crucial not just for the preservation of cultural heritage, but also for the prevention of biodiversity loss [26].

The use of medicinal plants is one of the elements of traditional knowledge that, because it is linked directly to health, is particularly sensitive for local communities [27]. It is estimated that 80% of the population in developing countries use medicinal plant resources for primary care [28]. Their use persists in rural and urban areas as a result of the transmission of knowledge, mostly in verbal form and between generations [29]. At the same time, the lack of access to public health services in rural areas incentivizes the use of medicinal plants [24, 25].

Despite their importance, knowledge of medicinal plants is subject to several threats due to, among other factors, urbanization [30, 31]. Urbanization leads to the loss of wild vegetation, reduction of the area dedicated to traditional agriculture, and transformation of natural areas for activities like commerce; at the same time, this contributes to cultural modification [32]. Land use change not only leads to the destruction of habitats of a variety of medicinal plants but also impacts the degree of knowledge of their management and uses. When medicinal plants no longer exist in the natural environment, the reflection on their use is also lost between one generation and the next [11, 33, 34]. Consequently, the use and management of medicinal plants could be modified by a reduction in the areas of collection and propagation, reluctance, and decrease in their use, as well as the perception of incompatibility between traditional and western medicine [27].

The general panorama of the effects of urbanization on the traditional knowledge of medicinal plants requires more research in order to clarify how certain factors associated with urbanization (access to official health services, migration, changing economic activities, etc.), affect the use of medicinal plants in traditional communities. It is important to document these processes in bioculturally megadiverse countries with a long tradition of use of medicinal plants and that currently face a scenario of loss of associated biocultural heritage due to, among other processes, urbanization.

The processes that deteriorate biocultural heritage are notorious in Mexico, one of the five most diverse countries worldwide [35], and where about 6,000 species of medicinal plants are used, of which at least 4,000 are collected from forests and jungles [36]. Despite this grand biocultural legacy resulting from thousands of years of interaction between diverse cultures and their environments [37], there are currently challenges that urgently need to be met. The country faces a public health emergency due to the obesity and diabetes epidemics, in addition to other diseases associated with sedentary lifestyle and increasing urbanization [38]. In addition, it is 43rd out of 194 countries in the rate of urbanization, with 80.2% of its inhabitants living in cities [39]. At the same time, it is ranked fifth worldwide in rates of deforestation [40], and land use change, including urbanization, has led to the destruction of ecosystems that harbor biodiversity, including species of medicinal plants.

In this study, we evaluate the knowledge of medicinal plants possessed by inhabitants of two communities with differing degrees of urbanization. At the same time, we explored the relationship between urbanization and the number of native and introduced species people knew, as well as sociocultural factors that influence species richness of medicinal plants used, comparing the relationships within and between communities.

This work was based on the premise that urbanization changes patterns of knowledge and use of medicinal flora, such that we expected inhabitants of the more urbanized community to know fewer species of medicinal plants and highlight introduced species, and that purchase would be the mode of acquisition of medicinal plants, all of which would suggest a loss of the knowledge of the local medicinal flora in this community. In contrast, we expected the less urbanized community to have more knowledge of local medicinal species, prefer native and wild species, and more frequently collect rather than purchase them.

## Methods

### Description of the study area

The study area is part of the Balsas river basin, a region in south-central Mexico from which most of the wild medicinal plants sold are extracted. This includes many markets and *tianguis* around the country, especially the Mercado de Sonora in Mexico City, which is perhaps the largest market for medicinal plants in Latin America [34, 41, 42]. In addition, this area has historically been a collection area for medicinal plants, in particular for many species that were used to pay tribute to Mexico-Tenochtitlan by the cultures and communities in the south of the state of Morelos [43]. Notable examples are *copal* (*Bursera* sp. [44];), *jícaras* (*Crescentia* sp. [45],) and *linaloe* (*Bursera linanoe* [46];). During the colonial period and until the last century, the area was an important collection area for medicinal plants, both in the Balsas River Basin (where tropical dry forest vegetation dominates), and in the Mixteca, a bioculturally diverse region which includes the three states of Guerrero, Puebla, and Oaxaca. The village of Tepalcingo is a very important settlement historically. It has pre-Hispanic roots, founded in 1272, and its inhabitants are widely known for their knowledge of the use and management of medicinal plants [47].

**Fig. 1:**
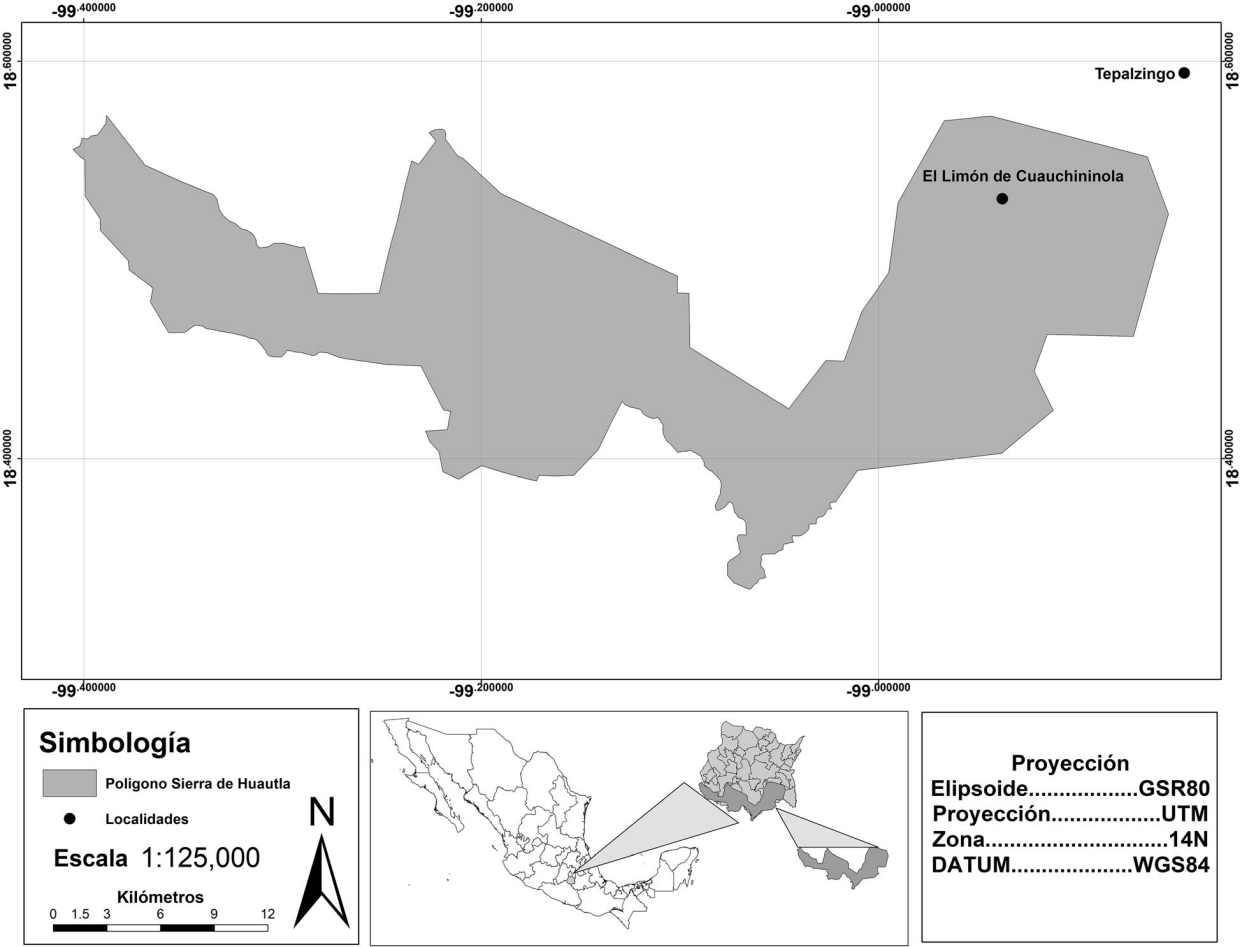
Sierra de Huautla Biosphere Reserve (*Reserva de la Biosfera Sierra de Huautla; REBIOSH*) and location of the study localities

The study area is near the border of the Sierra de Huautla Biosphere Reserve (*Reserva de la Biosfera Sierra de Huautla*; REBIOSH), in the state of Morelos, Mexico (Fig. 1). The reserve was decreed in 1999 and has an area of 59,030 hectares. It is covered almost entirely by TDF [48, 49], which is characterized by trees with an average height of 10 m that lose their leaves during the dry season [50]. The total population within the reserve is 25,356 inhabitants [51] in rural communities with high marginalization indexes due to little access to health, transportation, and education services and limited employment opportunities [52].

There are 939 species of vascular plants reported in the area, of which 602 (56%) are used by the communities to meet health, food, and shelter needs, among other uses [48]. About 400 species (66%) are medicinal plants that can be used to help resolve some health issues, since there are no public health services in 60% of the communities [48, 53].

We selected two communities—El Limón de Cuauchichinola (ELC) (within the REBIOSH) and Tepalcingo (TGO; in the area of influence of the REBIOSH)—which differ in their degree of urbanization (Table 1). ELC was founded in 1900 by a migrant population from other localities in the vicinity, and in 1929 was consolidated as an *ejido* (a mode of collective community-based land ownership in Mexico). ELC has a population of 129 and has a public primary school and local government house. A portion of its youth and adult population emigrate temporarily to the USA in search of employment opportunities. TGO is the municipal seat of the municipality of the same name and has a population of 12,053 inhabitants. It was founded in 1272 by native tribes, but it was not until 1869 that it was considered a municipality of Morelos. This community acts as a hub of distribution and trade in the region, and it is visited by people from different communities of southern Morelos to buy and sell products, which also makes it a destination for people who have migrated from other neighboring communities.

**Table 1:** Demographic data for the communities of El Limón de Cuauchichinola (ELC) and Tepalcingo (TGO)

Community/location	Number of inhabitants/no. of homes	Economic activities	Services	Average education (years)
ELC/18° 31′ 51″ N, 98° 56′ 15″ W, (1259 masl), 28.5 km from municipal seat	129/37	Seasonal agriculture, extensive livestock rearing, gathering of non-timber forestry products	37 homes in the community, all occupied, of which only 28 have electricity, running water, and public sewer lines. Public primary school and once monthly brigade offering free medical appointments	5.1
TGO/18° 35′ 47″ N, 98° 50′ 237″ W, (1160 masl). Municipal seat.	12,053/3674	Functions as a center for commerce and distribution of products and has approximately 370 different services, among which small shops and are the most frequent.	2382 homes have electricity, running water, and public sewer lines. Has education, public healthcare, and private medical practices, as well as wellness programs and public transportation	7.62

**Fig. 2:**
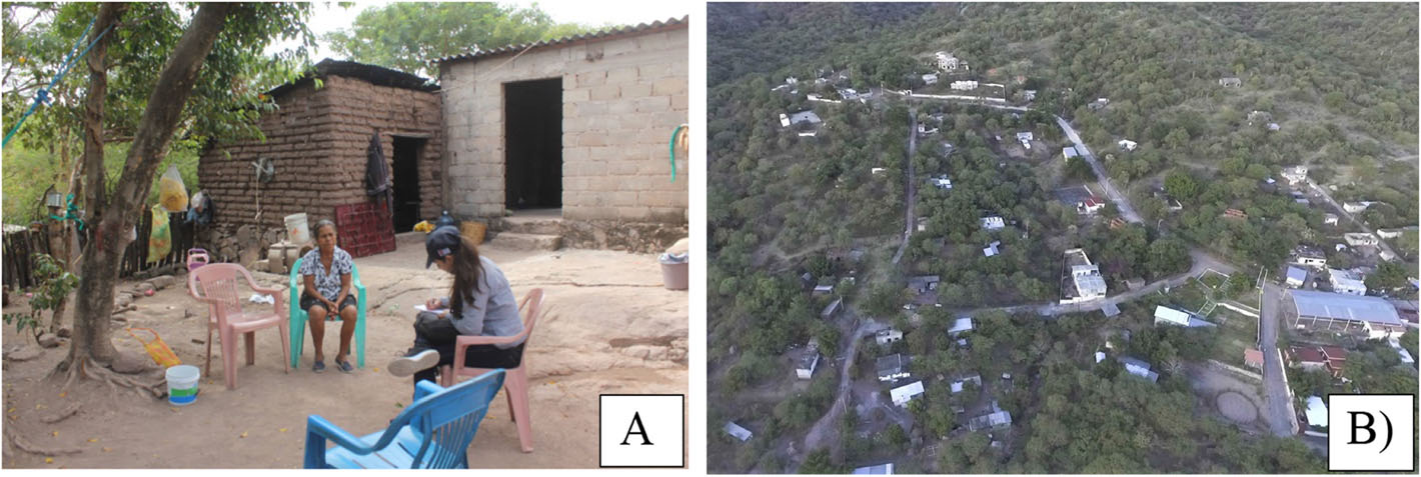
Community of El Limón de Cuauchichinola. **A** Interview with community members. **B** General panorama of the community. Photos: **A** C. Arjona, **B** Nextia multimedia

**Fig. 3:**
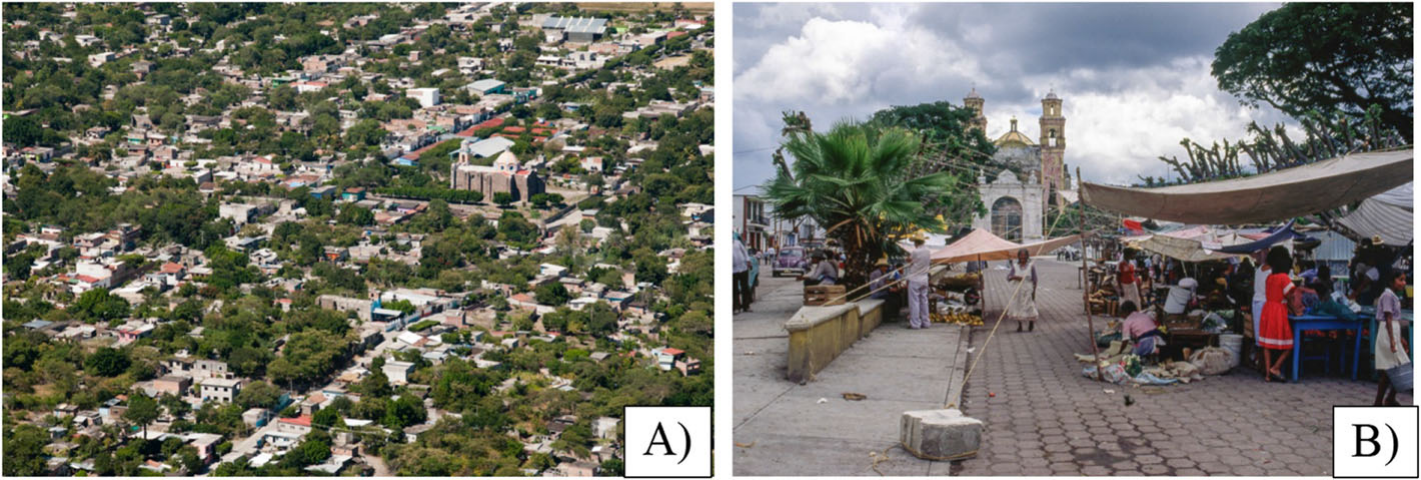
**A** General overview of the community of Tepalcingo. **B** Commercial activities in the center of the community. Photographs: **A** Sistema de Archivos Compartidos UAEM-3Ríos

The urbanization indicators that we used were the economic activities, availability of healthcare services, and average level of education. The socioeconomic data were obtained from the 2010 census [51] and the National Statistical Directory of Economic Units (*Directorio Estadístico Nacional de Unidades Económicas*) [52]. ELC is less urbanized than Tepalcingo, since its inhabitants depend almost exclusively on primary sector activities (Table 1, Fig. 2). TGO is a more urbanized community, since its inhabitants mainly work in the tertiary sector, such as commercialization and services (Table 1, Fig. 3).

### Stratified random sampling

In order to analyze the existence of an urbanization gradient that could impact knowledge of medicinal plants, we did stratified random sampling, differentiating regions within each community following the sampling design proposed by Pagaza [9]. We defined two regions—“central” and “peripheral” in each community. The central region referred to the area where the community’s administrative services were concentrated, while the periphery was defined as the areas near zones of agriculture, agroecosystems, and wild vegetation.

### Free listing and semi-structured interviews

We used free listing to document the number of medicinal plant species known by the inhabitants of each community, with 28 and 77 people in ELC and TGO respectively [54, 55]. We did a semi-structured interview [54] with the same people in order to collect personal data (name, age, sex, occupation, and birthplace), and data on medicinal plants, including their use, methods of preparation, parts used, method of acquisition (collection or purchase), conditions for which they are used, and if the interviewee had consulted with specialists in medicinal plants or traditional medicine. Saturation or redundancy of information was used to determine when the appropriate sample size was reached [56], and a non-parametric *t* test for unbalanced data was used in order to avoid biases in the results due to the difference in the total number of interviews per community.

### Structured interview

Using information from the stratified random sampling, we located 16 key informants (7 in ELC and 9 in TGO) who were recognized for their experience in the management of medicinal plants. We carried out structured interviews with these informants to obtain detailed information about the species of medicinal plants used, frequent ailments, plant parts used, method of acquisition, and opinions and perceptions concerning the persistence or erosion of the knowledge and use of medicinal plants [54].

### Ethnobotanical walks

To determine the taxonomic identities of the species recounted, both in the listing and in the structured interview, we carried out six ethnobotanical walks [57] in zones of wild and secondary vegetation, as well as agroecosystems in both communities (4 in TGO and 2 in ELC). The botanical specimens were collected and identified and deposited in the “HUMO” herbarium at the Center for Research in Biodiversity and Conservation (*Centro de Investigación en Biodiversidad y Conservación*, CIByC-UAEM).

### Quantitative analysis of information

In order to determine whether there were differences in the knowledge of medicinal plants between the two communities, we analyzed the results of the free listing and semi-structured interviews using Wilcoxon’s *W* test for samples with asymmetrical distribution. This analysis was done for the total number of species named, then separately for the number of native, introduced, and wild species mentioned. The differences were evaluated between communities, regions, occupations, sex, and birthplace of the interviewee. The analyses were done in SPSS software, version 24.0 [58].

Using the data from the free listing and the semi-structured interviews, we constructed a database with 16 variables that considered the socioeconomic information and degree of knowledge of medicinal plants in the interviewed populations of the two communities. In order to characterize the differences in knowledge of medicinal plants depending on the degree of urbanization within and between the communities, we did a discriminant function analysis using SPSS software, version 24.0 [58].

### Qualitative analysis of information

We did a qualitative analysis of the information from the interviews with key informants in order to characterize the ideas, comments, and perceptions associated with knowledge of medicinal plants. This approach from the social sciences guides the research question, allowing for deep exploration of the changes in knowledge of medicinal plants from the perspective of people from the localities who have broad experience with their management [59]. This methodology is based on the notion that reality is socially constructed, and that people therefore give meaning to social and natural phenomena according to their perceptions of the world [60–62]. The interviews from the two communities were transcribed and codified using the program ATLAS.ti version 7.5 [63], organizing the information according to the perceptions of the key informants into four coded categories: treatment preferences, teaching-learning, availability of medicinal plants, and problems. The codification of the information consisted in an exploratory line-by-line reading and selection of particular data in order to reduce the information into a format that was manageable for analysis and interpretation. We also created a perception map linking the responses obtained and enumerating the responses that were similar among interviewees [64–67]. This map was included because it serves as a graphical summary of the different perceptions and helps structure the narrative of the results and discussion.

## Results

In the two communities studied, we recorded a total of 269 common names of medicinal plants, which correspond to 217 species, of which 148 (68%) are native to Mexico, 79 (36%) are naturally distributed in the study area, and 69 (31%) are introduced. The total richness was grouped into 70 botanical families, and the families with the largest number of species were Fabaceae, (28 species), Asteraceae (21), Lamiaceae (11), Solanaceae (9), and Malvaceae (8).

### Differences in the degree of knowledge of medicinal plants by community

**Fig. 4:**
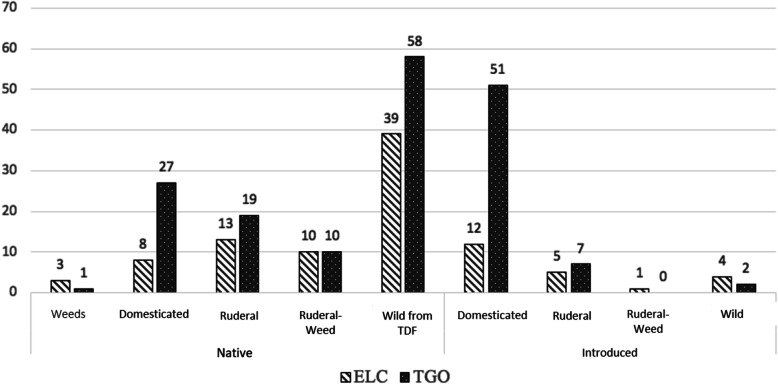
Number of species mentioned in each community, categorized by plant origin and degree of management

In ELC, 95 species of medicinal plants were mentioned, distributed in 46 botanical families, of which 73 are native to Mexico (42%), 39 are considered part of the tropical dry forest (51%), and 22 are introduced (12%) (Fig. 4). In TGO, 175 species of medicinal plants were named, which are distributed in 71 botanical families, 115 are native to Mexico (66%), 60 species are introduced (34%), and 58 are tropical dry forest species (Fig. 4).

Wilcoxon’s *W* test showed significant differences between the communities (ELC vs. TGO) at all levels of the analysis of knowledge of medicinal plants (total number of species mentioned and number of native, introduced, and wild species). The region factor showed differences between the center and periphery of the two communities in the total number of species, the number of native species, and the number of wild species, but not the number of introduced species. Women mentioned more introduced species than men (*W =* 1,314, *p =* 0.002), with no differences in the total, native, or wild species (Table 2). People that worked in the field mentioned more wild species than homemakers (*W =* 704, *p =* 0.016). There were no significant differences when comparing among birthplaces (Table 2).

**Table 2:** Results of Wilcoxon’s *W* test of differences in knowledge of medicinal plants between communities (*ELC*, El Limón de Cuauchichinola; *TGO*, Tepalcingo). Bold text indicates significant differences (*P* < 0.01)

	Community		Region		Occupation		Sex		Birthplace	
	Total number of species named									
	ELC	TGO	Center	Periphery	Primary activities	Homemakers	Female	Male	In the study communities	In other communities in Morelos
W	**1885.50**		**2278.00**		612.00		1568.50		763.00	
N	**0.003**		**0.0399**		0.3921		0.2111		0.2682	
Total number of native species named										
W	**1212.50**		**2209.00**		656.50		1923.50		811.50	
P	**0.0460**		**0.0123**		0.1099		0.2265		0.5113	
Total number of introduced species named										
W	**2080.50**		2680.50		489.50		**1314.00**		797.50	
P	**0.0001**		0.5876		0.2336		**0.0024**		0.4279	
Total number of wild species named										
W	**22050.00**		**2243.50**		**704.00**		1960.00		827.00	
P	**0.0001**		**0.0220**		**0.0166**		0.1417		0.6067	

### Knowledge of medicinal plants and urbanization gradient

**Fig. 5:**
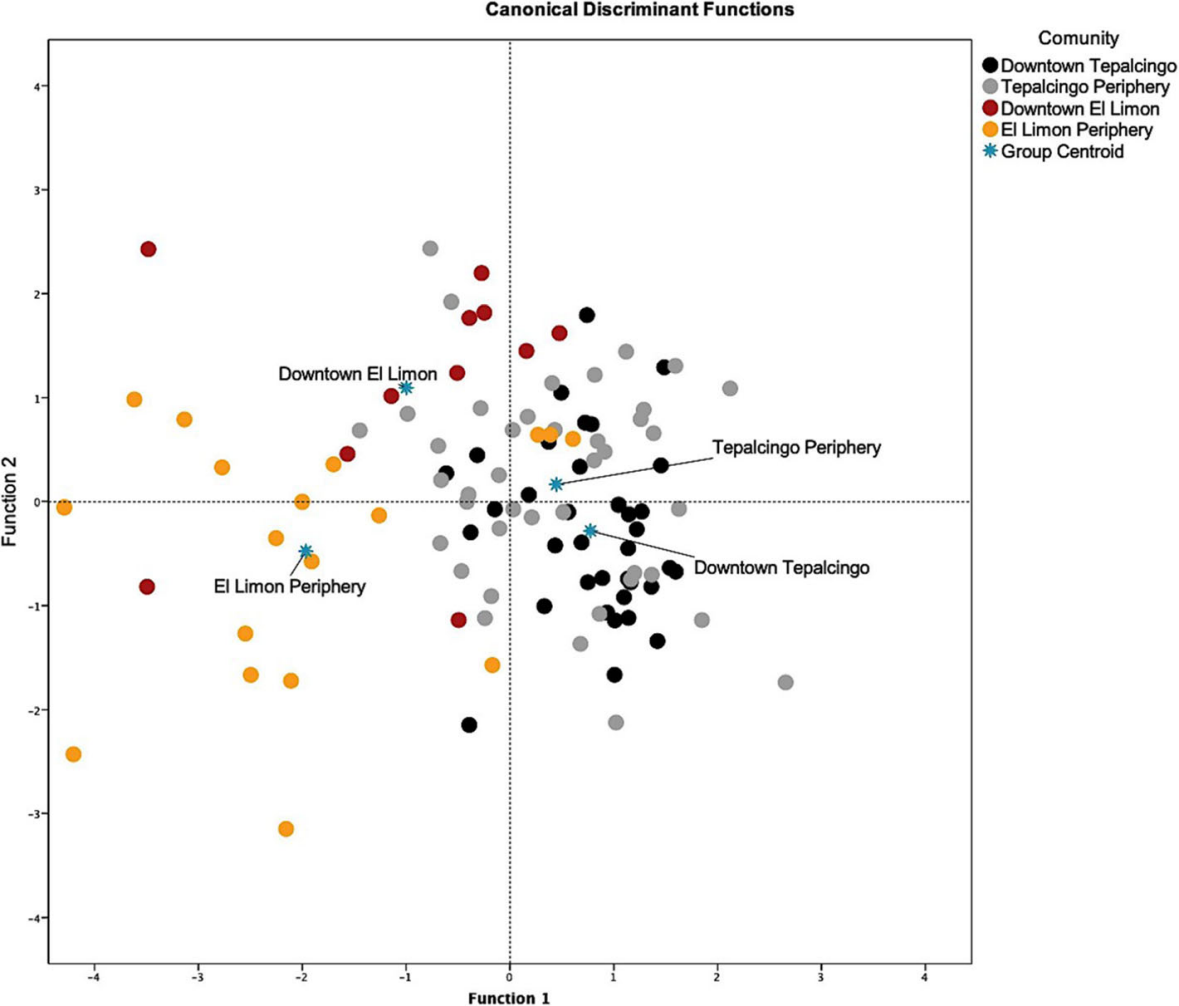
Distribution of interviewees according to degree of urbanization

The discriminant function analysis showed that people’s knowledge of medicinal plants was affected by urbanization. As shown in Table 3, the first two functions explained 92% of the variation, with the first explaining 77.2% and the second 14.8%. The grouping of the interviewees in discriminant function 1 was statistically significant, which was also confirmed by the canonical correlation value and Wilk’s lambda. Figure 5 shows that the interviewees were distributed along an urbanization gradient, in which the periphery of ELC is shown in the yellow oval on the left-hand side of the graph, followed by interviewees from the central zone of ELC (red oval). The distribution of the interviewees from the periphery of TGO (gray oval) and from the center of TGO (black oval) were interspersed with each other. The most important variables in Function 1 (Table 4) were age of informants, number of spp. collected, total spp. recorded in our research, number of native spp. mentioned, total native spp. recorded, number of wild spp. reported, and the use of the species. These all had a negative sign, which means that people located toward the right-hand side of the graph (TGO) had on average lower age, referred to a lower number of species collected, and a lower number of native and wild species compared to people located on the left-hand side of the graph (ELC). Variables with positive values were the number of species purchased, domesticated, and introduced species. Thus, interviewees located on the right-hand side of the graph reported, on average, purchasing medicinal plants more frequently, and using a larger number of domesticated and introduced species.

**Table 3:** Autovalues and Wilk’s *l*ambda from the discriminant functions analysis, using as a grouping variable the center and periphery regions of each study community

Function	Autovalue	% variance	Cumulative %	Canonical correlation
1	1.081	77.2	77.2	0.721
2	0.208	14.8	92.0	0.415
Test of functions	**Wilk’s lambda**	**Chi-squared**	**df**	**Sig.**
1 to 2	0.358	98.658	42	0.000
2	0.745	28.301	26	0.344

**Table 4:** Relative importance of the variables studied in the first two discriminant functions

Standardized canonical discriminant function coefficients		
**Variable**	**Function**	
	**1**	**2**
Sex	− 0.1002	− 0.0953
Age	**− 3.1193**	6.4347
Birthplace	− 0.1734	0.1926
Ocupation	0.3962	0.3197
Number of species	− 0.7418	− 0.6630
Number of native species	**− 1.4697**	− 0.0436
Number of introduced species	0.7278	− 0.6194
Number of wild TDF species	**− 1.2784**	0.3881
Number of domesticated species	**1.0076**	− 1.0529
Number of ruderal species	− 0.1274	− 0.1139
Number of herbaceous species	− 0.0989	− 0.1353
Number of ruderal and herbaceous species	− 0.3304	0.0692
Number of species collected	**− 1.7439**	− 0.5615
Number of species purchased	**1.0749**	− 0.0085
	0.3174	− 0.1511

Bold text indicates the most important variables in the discriminant function

Table 5 shows that 61.3% of the total interviewees were correctly classified according to the degree of urbanization assigned to each community. The majority of interviewees were correctly classified as inhabitants of the periphery of ELC (77.8% assigned correctly), the center of TGO (63.9%), the periphery of TGO (56.1%), or the center of ELC (45.5%).

**Table 5:** Classification of interviewees according to the urbanization gradient in the study communities. The data show raw and percentage values [33, 36]

Comunidad	Predicted group membership				Total
	Tepalcingo Center	Tepalcingo Periphery	El Limón Center	El Limón Periphery	
Tepalcingo Center	23	13	0	0	36
Tepalcingo Periphery	15	23	2	1	41
El Limón Center	1	3	5	2	11
El Limón Periphery	4	0	0	14	18
Tepalcingo Center	63.9%	36.1%	0%	0%	100%
Tepalcingo Periphery	36.6%	56.1%	4.9%	2.4%	100%
El Limón Center	9.1%	27.3%	45.5%	18.2%	100%
El Limón Periphery	22.2%	0%	0%	77.8%	100%

61.3% of original grouped cases correctly classified

### Perceptions and qualitative analysis of knowledge of medicinal plants in different urbanization contexts

In ELC, seven key informants were interviewed—two men and five women between 54 and 73 years of age. Most of these people were born in the community and their occupation was in the primary sector (agriculture, livestock, gathering) and in the home. These people preferred to use medicinal plants to cure illness, since these are abundant in their communities and are a free alternative for the treatment of many ailments. However, they mentioned that if the use of these plants does not lead to improvement, or with specific conditions such as bites/stings or severe illness, they must travel to another more urban community (63-km distance, almost a 2-h journey) to seek treatment at a health clinic that is open all year round which offers services that are not available in the community; this travel has time and monetary costs.

**Fig. 6:**
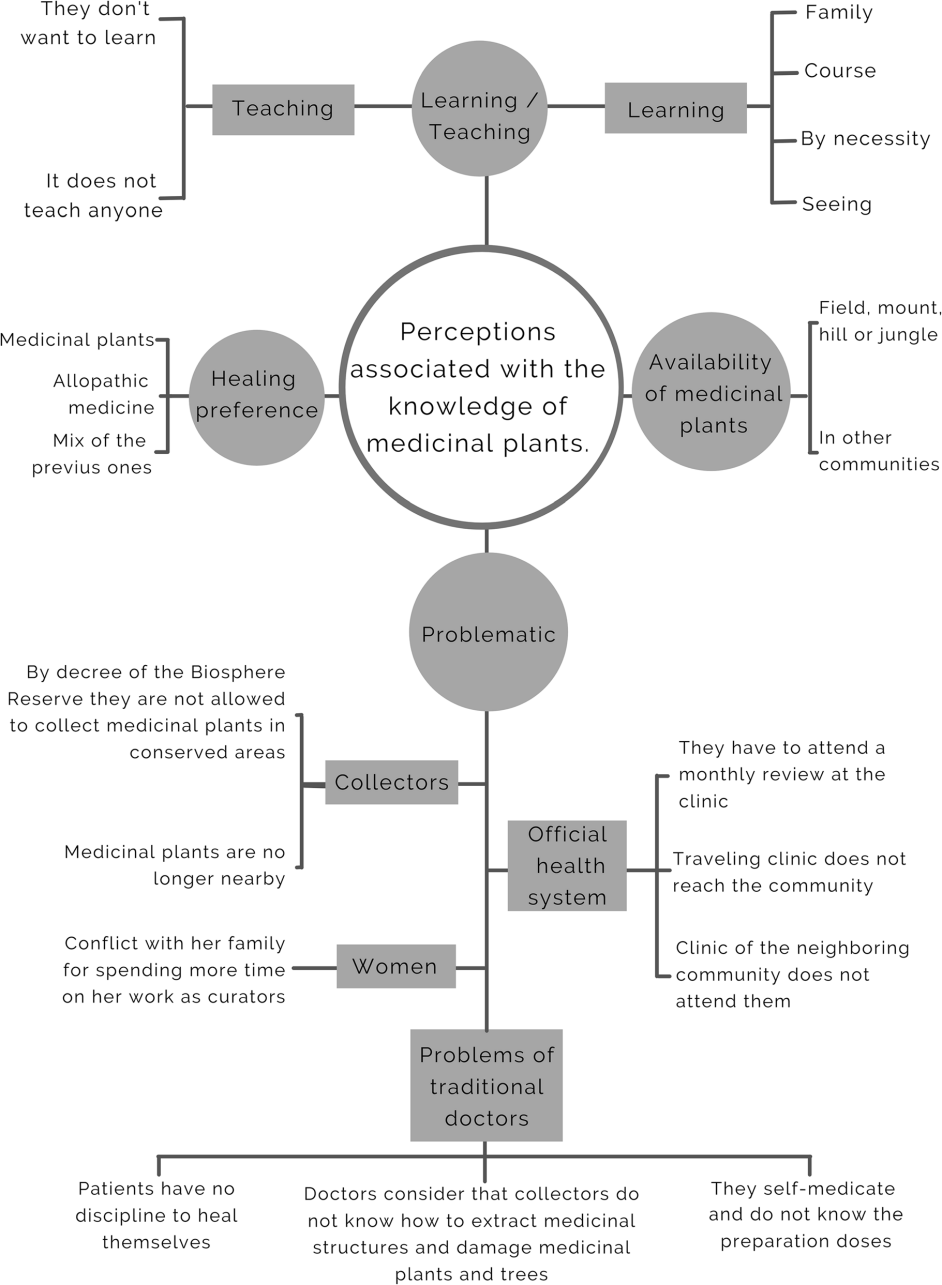
Perception map based on interviews with key informants from ELC and TGO

The majority of the interviewees in ELC learned to use medicinal plants directly from family members or by observing their use by other people (4). However, none had transmitted their own knowledge to others, and they mentioned that knowledge of medicinal plants is being lost since inhabitants prefer the speed of allopathic medicine, because people are no longer interested in these plants or do not consider them effective, or because they are lazy or lack the discipline to prepare the remedies. The key informants from ELC did not consider themselves traditional medical practitioners, but the population did recognize them as experts and occasionally consult them on their knowledge of medicinal plants, such that they did play an important role in the community (Fig. 6). The ELC interviewees mentioned 81 ailments that are cured with medicinal plants (Table 6). The most frequently mentioned were stomachache (30 mentions), diarrhea and wounds (25 each), cystitis (13), inflammation (12), and gastritis and postpartum ailments (11 each). The most mentioned methods of preparation were by boiling the plant to make teas or infusions to drink with 58 species, followed by the cleaning of wounds with the infusions of 12 species (Table 7).

**Table 6:** Average number of species mentioned by occupation

Occupation	TGO		ELC	
	Average	Standard deviation	Average	Standard deviation
**Primary sector**	7.66	3.01	10.06	4.93
**Homemaker**	10.625	6.13	9.53	6.38

**Table 7:** All recorded plants and their uses

***Species***	Common name	Origin (1: native, 2: introduced)	Number of mentions (ELC)	Number of mentions (TGO)	Use	Preparation methods
*Acacia angustissima* (Mill.) Kuntze	Timbre	1	5	4	Stomachache, acne	Tea, machacado
*Acacia bilimekii* J.F.Macbr.	Tehuixtle	1	0	2	Cold sore, bruxism	Chewed
*Acacia farnesiana* (L.) Willd.	Huizache	1	1	6	Cold sore, bruxism, kidney stones, prostate, liver	Tea, applied to affected area, placed with salt, chewed, smeared
*Acalypha aristata* Kunth	Hierba del cancer	1	1	0	Wounds, diuretic, kidneys, gastric ulcer	Tea
*Acosmium panamense* (Benth.) Yakovlev	Chichiqui	1	0	1	Psoriasis	Washing
*Agave angustifolia* Haw.	Maguey	1	0	1	Wounds	Drops over wound
*Agave salmiana* Otto ex Salm-Dyck	Maguey de Pulque	1	0	1	Cancer prevention	Tea
*Allium cepa* L.	Cebolla	2	0	4	Diabetes, cough, teething pain in babies, tonsillitis	Placed on feet, tea
*Allium sativum* L.	Ajo	2	1	6	Cough, diabetes, teething pain in babies, scorpion sting	In alcohol, eaten, tea
*Aloe vera* (L.) Burm.f.	Sábila	2	3	28	Prostate, promote weight loss, kidneys, gastritis, wounds, diabetes, menstrual cramps, gastric ulcer, inflammation, skin burns, hair health, postpartum, high blood pressure, kidneys, stomachache, cancer	Liquified, drink with milk, placed on feet, juice, patches, applied to affected area, eaten, grilled, smeared, baths
*Amphipterygium adstringens* (Schltdl.) Standl.	Cuachalalate	1	23	60	Wounds, gastritis, kidneys, cancer, inflammation, lungs, rash, kidney stones, ulcers, menstrual cramps, stomachache, diarrhea	Boiled, place dried and ground on area with pain, washing, baths, tea, wine
*Ananas comosus* (L.) Merr.	Piña	2	0	3	Diabetes, uric acid, kidney stones	Liquified
*Annona muricata* L.	Guanábana	2	0	6	High blood pressure, cancer, diabetes	Tea, eaten
*Apoplanesia paniculata* C.Presl	Escobilla	1	0	1	Diarrhea	Tea
*Aristolochia subclausa* S.Watson	Tlacopatle	1	0	1	Foot pain	Applied to affected area
*Artemisia absinthium* L.	Ajenjo	1	0	2	Stomachache, bile	Tea
*Artemisia ludoviciana subsp. mexicana* (Willd. ex Spreng.) D.D.Keck	Estafiate	1	0	4	Stomachache, diarrhea	Tea
*Artocarpus heterophyllus* Lam.	Yaca	2	0	1	Anemia	Tea
*Arundo donax* L.	Cañaveral/Carrizo	2	2	2	Kidneys, lungs, pain	Boiled, tea
*Asclepias curassavica* L.	Venenillo, algodoncillo	1	1	1	Cold sores	Applied to affected area
*Asterohyptis stellulata* (Benth.) Epling	Chia	1	0	1	Diarrhea	Tea
*Avena sativa L.*	Avena	2	0	3	Blood pressure	Liquified, tea
*Azadirachta indica* A. Juss.	Neem	2	1	23	Diabetes, kidneys, pain in hands, circulation, stress	Boiled, capsules
*Barkleyanthus salicifolius* (Kunth) H.Rob. & Brettell	Azumiate	1	0	3	Energetic cleansing	Rubbed on body
*Bidens bipontina* Sherff	Acetillo	1	0	0	Wounds	Smeared
*Bidens odorata* Cav.	Aceitillo	1	2	1	Wounds	Applied to affected area
*Bougainvillea glabra* Choisy	Bugambilia	2	2	15	Cough, rash, colds	Boiled, tea, baths
*Bouvardia ternifolia* (Cav.) Schltdl.	Clavillo	1	3	0	Kidneys, diabetes	Tea
*Brassica smelledacea* L.	Col	2	0	2	Indigestion, insomnia	Placed under the pillow belly and back
*Bromelia pinguin* L.	Tim	1	0	0	Kidneys, gastritis	Tea, eaten
*Brownea ariza* Benth.	Palo de Cruz	2	2	0	Bruises	Washing, tea
*Bunchosia canescens (Aiton) DC.*	Nanche criollo/Nanche de perro	1	1	1	Inflammation, diarrhea	Tea
*Bursera ariensis* (Kunth) McVaugh & Rzed.	Palo de Oro/Palo Dorado	1	13	3	Bones, fever, knees, heels, hips, fractures	Applied to affected area, patches, tea
*Bursera bipinnata* (Moc. & Sessé ex DC.) Engl.	Copal de Piedra	1	0	1	Postpartum	Smoked
*Bursera copallifera* (Sessé & Moc. ex DC.) Bullock	Copal	1	4	0	Cough, purify blood, fever	Inhale steam, patches, boiled
*Bursera grandifolia* (Schltdl.) Engl.	Palo mulato	1	1	2	Wounds, bruises, purify blood	Boiled, smeared, washing
*Byrsonima crassifolia* (L.) Kunth	Nanche	1	0	1	Indigestion	Tea
*Calea zacatechichi* Schltdl.	Prodigiosa/Zacatechichi/Amula	1	20	27	Diabetes, anger, terror, stomachache, menstrual cramps, diarrhea, cold, diabetes	Tea, alcohol
*Camellia sinensis* (L.) Kuntze	Tea de la india	2	0	0	Anger	Boiled
*Cannabis sativa* L.	Marihuana	2	0	1	Muscle pain	Alcohol
*Capsicum annuum* L.	Chile	1	0	1	Aerophagia in children	Smoked
*Carica papaya* L.	Papaya/Papayo	1	1	5	Scorpion sting, stomachache, dengue, Zika	Tea, baths, applied to affected area
*Carya illinoinensis* (Wangenh.) K.Koch	Nuez	2	0	1	Diabetes	Liquified
*Cascabela ovata* (Cav.) Lippold	Ayoyote	1	1	0	Skin infections	Tea
*Casimiroa edulis* La Llave	Zapote blanco	1	0	4	High triglycerides, high blood pressure, insomnia, cough	Tea, placed under the pillow
*Cecropia obtusifolia* Bertol.	Guarumbo	1	0	1	Diabetes	Tea
*Ceiba pentandra* (L.) Gaertn.	Pochote	1	0	1	Strenghten immune system	Tea
*Chamaemelum nobile* (L.) All.	Manzanilla	2	0	54	Stomachache, eye irritation, flu, colic in babies, fever, diarrhea, cough	Tea, drops, washing
*Chamaesyce umbellulata* (Engelm. ex Boiss.) Millsp.	Hierbabuenilla	2	0	0	Bruises and inflammation	Tea
*Chelidonium majus* L.	Celidonea	2	0	2	Skin rash, inflammation	Baths, applied to affected area
*Chiranthodendron pentadactylon* Larreat.	Flor de Manitas	1	0	1	Pain	Tea
*Cinnamomum verum* J.Presl	Canela	2	0	7	Cough, diarrhea, vomit, stomachache	Tea
*Cirsium ehrenbergii* Sch.Bip.	Cardo	1	0	1	Love potion	Alcohol
*Cissus verticillata* (L.) Nicolson & C.E.Jarvis	Tripa de Judas	1	2	0	Scorpion sting	Boiled
*Citrus aurantiifolia* (Christm.) Swingle	Lima/Lima Chichona/Lima Real/Flor de Azahar	2	0	5	High blood pressure, nerves	Tea, eaten, liquified
*Citrus limon* (L.) Osbeck	Limón/Flor de Azahar	2	1	16	Nerves, prostate, diarrhea, low blood pressure, kidney stones, inflammation, flu, diabetes, fever, Scorpion sting, cough, chest pain, insomnia	Tea, necklace, liquified, juice, rinses, applied to affected area, with milk
*Citrus maxima* (Burm.) Merr.	Toronja	2	0	1	High triglycerides	Juice
*Citrus reticulata* Blanco	Mandarina	2	0	1	Cough	Tea
*Citrus sinensis* (L.) Osbeck	Naranja/Flor de Azahar	2	1	3	Postpartum, flu, cough, headache	Boiled, juice, baths
*Cnidoscolus aconitifolius* (Mill.) I.M.Johnst.	Chaya	1	0	3	Prostate, uric acid, kidneys	Tea, liquified
*Cochlospermum vitifolium* (Willd.) Spreng.	Palo de Pánico/Pánico	1	4	0	Diabetes, gastritis	Tea
*Cocos nucifera* L.	Coco	2	0	1	Platelets	Tea
*Coffea arabica* L.	Café	2	0	1	Dengue	Placed on soles with butter
*Commelina zebrina* C.B.Clarke	Hierba del pollo/Hoja de pollo	1	0	7	Wounds, diabetes, dysentery, anemia, kidneys	Tea, liquified
*Conyza coronopifolia Kunth*	Gordolobo	1	2	1	Cough, bronchitis	Tea
*Cordia boissieri* A.DC.	Anacahuite	1	2	2	Cough	Tea
*Cordia morelosana* Standl.	Palo Prieto	1	0	2	Cough, cystitis	Tea
*Coryphantha elephantidens subsp. bumamma* (Ehrenb.) Dicht & A.Lüthy *	Biznaga	1	2	2	Diabetes, pityriasis rosacea, kidneys	Tea, liquified, eaten
*Costus spicatus* (Jacq.) Sw.	Caña	1	0	1	Kidneys	Tea
*Crataegus mexicana* DC.	Tejocote	1	0	1	Cough	Tea
*Crescentia alata* Kunth	Cuatecomate	1	14	50	Cough, kidneys, asthma, bronchitis, pneumonia, lungs, cancer, gastric ulcer, earache	Drops, tea, syrup, alcohol, wine
*Croton niveus* Jacq.	Copalzi	1	1	0	Purify blood, diabetes	Boiled
*Cucumis sativus* L.	Pepino	2	0	1	Wounds	Applied to affected area
*Cucurbita moschata* Duchesne	Calabaza	1	1	0	Diarrhea	Tea
*Cuminum cyminum* L.	Comino	2	0	4	Stomachache, bulging belly, vomiting, diarrhea	Tea
*Cunila lythrifolia* Benth.	Poleo	1	0	3	Flu, pain	Vaporizations, tea
*Cuphea micropetala* Kunth	Taray	1	0	1	Kindeys	Tea
*Cuscuta tinctoria Mart. ex Engelm.*	Chahuistle	1	0	1	Cold sores, sores	Applied to affected area
*Cymbopogon citratus* (DC.) Stapf	Tea de Caña/Zacatelimón	2	0	3	Nerves, blood pressure	Tea
*Dalea alopecuroides* Willd.	Escoba	1	0	2	Vaginal bleeding	Tea
*Datura innoxia* Mill.	Toloache	1	1	0	Swollen pimples, bruises	Tea
*Diphysa americana* (Mill.) M.Sousa	Chicharronsillo	1	1	0	Fever, diarrhea	Tea
*Dysphania ambrosioides* (L.) Mosyakin & Clemants	Epazote	1	1	10	Diarrhea, stomach parasites, menstrual cramps, stomachache	Tea, eaten
*Elytraria acaulis* (L.f.) Lindau	Viborilla	2	3	0	Snakebite or scorpion sting	Boiled, chewed
*Equisetum arvense* L.	Cola de caballo	1	0	5	Kidneys, wounds, cystitis	Tea
*Eriobotrya japonica* (Thunb.) Lindl.	Níspero	2	0	1	Migraine	Tea
*Eryngium beecheyanum* Hook. & Arn.	Hierba del sapo	1	0	1	Swollen feet	Tea
*Erythrina americana* Mill.	Chompantle	1	1	0	Kidneys	Tea
*Eucalyptus globulus* Labill.	Eucalipto	2	1	7	Flu, bronchitis	Tea, vaporizations
*Euphorbia aaron-rossii* A.H.Holmgren & N.H.Holmgren	Istumeca	1	0	1	Fever	Applied to affected area
*Euphorbia tithymaloides* L.	Zapatito	1	0	2	Buried spines	Applied to affected area
*Eysenhardtia polystachya* (Ortega) Sarg.	Palo Dulce/Palo Azul	1	15	20	Kidneys, cystitis, diabetes, burning feet, gastritis	Tea, washing,
*Ficus carica* L.	Higo	2	0	3	High triglycerides, diabetes, wounds	Tea
*Ficus sp.*	Amate	1	0	2	Fever	Placed on feet
*Foeniculum vulgare* Mill.	Hinojo	2	0	1	Stomachache, diarrhea	Tea
*Guadua amplexifolia* J.Presl	Otate	1	0	1	Gumboil	Tea
*Guazuma ulmifolia* Lam.	Caulote/Cuauhulote	1	0	5	Red stains in babies, kidneys, colic in babies	Baths, washing, infusion
*Haematoxylum brasiletto* H.Karst.	Palo de Brasil	1	14	15	Prostate, kidneys, cystitis, herpes, clean face, fever, diabetes, lungs, blood pressure	Tea, rinses
*Helianthemum glomeratum* (Lag.) Lag. ex Dunal	Santa Martha	1	0	1	Muscle ache	Alcohol
*Heliocarpus americanus* L.	Clanguilagua	1	0	2	Cystitis, Scorpion sting	Tea, eaten
*Heteropterys brachiata* (L.) DC.	Margarita	1	3	3	Skin irritation, purify blood, cough, kidneys	Tea, baths, infusion
*Heterotheca inuloides* Cass.	Árnica	1	6	13	Sprains, wounds, gastritis, kidney stones, pain, bruises, inflammation, liver	Tea, plater, applied to affected area, washing, smeared, poultice
*Hibiscus sabdariffa* L.	Jamaica	2	0	1	Kidneys	Tea
*Hintonia latiflora* (Sessé & Moc. ex DC.) Bullock	Quina	1	1	2	Bile, wounds, flu, purify blood	Tea
*Hippocratea excelsa* Kunth	Ixcate/Cancerina	1	3	7	Wounds, gastritis, yeast infections, cold sores	Tea, ground, washing, applied to affected area
*Illicium verum* Hook.f.	Anís/Anís de Estrella	2	0	3	Stomachache, vomiting, diarrhea	Tea
*Inga vera* Willd.	Jinicuil	1	1	0	Stress	Tea
*Ipomoea arborescens* (Humb. & Bonpl. ex Willd.) G. Don	Palo Blanco/Cazahuate	1	1	2	Cancer, scorpion sting	Tea
*Ipomoea murucoides* Roem. & Schult.	Cazahuate amarillo	1	1	1	Throat inflammation, cough	Tea, applied to affected area
*Justicia spicigera* Schltdl.	Muicle	1	2	15	Kidneys, rash, varicella, red blood cells, circulation, migraine, cystitis, allergies	Baths, tea,
*Lasiacis nigra* Davidse	Carricillo	2	0	3	Kidneys, gallbladder	Liquified, tea
*Lemaireocereus hollianus* (F.A.C. Weber) Britton & Rose	Calehuale	1	1	0	Diarrhea	Tea
*Leucaena esculenta* (DC.) Benth.	Guaje colorado	1	0	1	Cold sore	Applied to affected area
*Lippia dulcis* Trevir.	Hierba Dulce	1	1	0	Head ache	Boiled
*Lippia graveolens* Kunth	Orégano	1	0	3	Stomachache, menstrual cramps	Tea
*Loeselia mexicana* (Lam.) Brand	Espinosilla	1	1	0	Fever	Tea
*Ludwigia octovalvis* (Jacq.) P.H.Raven	Clavillo	1	0	1	High blood pressure	Tea
*Malus domestica* Borkh.	Manzana	2	0	1	Diabetes	Liquified
*Malva parviflora* L.	Malva	2	0	0	Inflammation	Tea
*Malvaviscus arboreus* Cav.	Monacillo	1	0	1	Stomachache	Tea
*Mangifera indica* L.	Mango	2	0	2	Diabetes, cough	Tea
*Marginatocereus marginatus* (DC.) Backeb.	Orégano	1	1	1	Scorpion sting	Applied to affected area
*Marina nutans* (Cav.) Barneby	Escoba roja	1	0	0	Vaginal bleeding, fidelity	Tea
*Marrubium vulgare* L.	Manrubio	2	3	0	Diarrhea	Boiled
*Marsdenia lanata* (Paul G. Wilson) W.D. Stevens	Caxancapatli blanco	1	0	1	Postpartum	Baths
*Melia azedarach* L.	Paraíso	2	0	1	Indigestion	Tea
*Mentha canadensis* L.	Hierba buena	2	12	42	Stomachache, vomit, menstrual cramps, diarrhea, toothache, stress, bad breath, bruises	Tea, foments, infusion, chewed
*Mentha spicata L.*	Menta	2	0	1	Stomachache	Tea
*Microlobius foetidus* (Jacq.) M.Sousa & G.Andrade	Hediondillo	1	0	1	Rash in children	Rub over body
*Mimosa affinis* Robinson	Dormilona	1	0	0	Vaginal bleeding	Tea
*Mimosa albida* Willd.	Uña de Gato	1	0	2	Kidneys, prostate	Tea
*Mimosa benthamii* J.F.Macbr.	Tecolhuixtle	1	1	0	Wounds	Tea
*Montanoa tomentosa* Cerv.	Vara de Cuilota	1	0	1	Bruises	Washing
*Morinda citrifolia* L.	Noni	2	0	2	High triglycerides, diabetes	Tea, liquified
*Moringa oleifera* Lam.	Moringa	2	1	26	Diabetes, menstrual cramps, dizziness, knee pain, kidneys, cancer, high triglycerides, energy	Tea, liquified, eaten, chewed
*Moussonia deppeana* (Schltdl. & Cham.) Klotzsch ex Hanst.	Cacahuetillo	1	1	0	Diarrhea	Tea
*Musa balbisiana* Colla	Platano macho	2	1	0	Diabetes, gastritis	Tea
*Myristica fragrans* Houtt.	Nuez Moscada	2	0	4	Lungs, anger, dizziness, blood pressure, postpartum	Wine, placed under tongue or area with pain, smoked
*Myroxylon balsamum* (L.) Harms	Guayacán amarillo	2	4	0	Anger	Boiled
*Nicotiana glauca* Graham	Hojas de gigante	1	0	1	Toothache	Smeared
*Nicotiana tabacum* L.	Tabaco	1	0	2	Postpartum, fever, chills	Baths, applied to affected area
*Nissolia fruticosa* Jacq.	Manea de toro	1	0	1	Kidneys	Tea
*Ocimum basilicum* L.	Albahaca	2	3	10	Bad air, cold, cough, inflammation, stomachache, earache, eye cataracts	Baths, inhale steam, tea, applied to affected area, placed in eyes, smeared
*Oenothera rosea* L'Hér. ex Aiton	Hierba del golpe	1	4	6	Bruises, muscle aches, gastric ulcer, sprains, inflammation	Applied to affected area, smeared, foments, tea, washing, plater
*Opuntia atropes* Rose	Nopal	1	1	2	Inflammation, diabetes, kidneys	Tea, liquified
*Parmentiera aculeata* (Kunth) Seem.	Cuajilote	1	0	2	Kidneys, earache	Eaten, drops, tea
*Parthenium hysterophorus* L.	Cola de zorra	1	2	0	Anger	Tea
*Passiflora edulis* Sims	Maracuyá	2	0	26	Cough, dengue, platelets, diabetes, blood pressure	Tea, syrup, juice
*Peperomia granulosa* Trel.	Gordoncillo	1	1	0	Bone fractures	Tea
*Persea americana* Mill.	Aguacate	1	0	1	Menstrual cramps	Tea
*Persea liebmannii* Mez	Aguacatillo	1	1	0	Tapeworms	Tea
*Phalaris canariensis L.*	Alpiste	2	0	3	Diabetes, blood pressure	Tea, savor, applied to affected area, massage, baths
*Physalis pubescens* L.	Tomate	1	0	7	Tonsillitis, indigestion, vomit, postpartum, narrow palate	Poultice
*Pilea pubescens* Liebm.	Chichicastle	1	0	1	Skin allergies	Baths
*Pinus leiophylla* Schiede ex Schltdl. & Cham.	Ocote	1	0	1	Cough	Tea
*Piper auritum* Kunth	Hoja santa	1	0	1	Menstrual cramps	Tea
*Piper leucophyllum* (Miq.) C. DC.	Cordoncillo	1	0	1	Purify children	Rub over body
*Pithecellobium dulce* (Roxb.) Benth.	Guamuchil	1	1	1	Cystitis	Tea
*Pithecellobium sp.*	Guamuchil morado	1	0	1	Wounds	Washing
*Plectranthus hadiensis* (Forssk.) Schweinf. ex Sprenger	Hierba de Vaporub/Alcanfor	2	0	3	Cold, wounds, cough	Smeared, infusion, tea
*Polygonum aviculare* L.	Lengua de pajaro	1	0	1	Cystitis	Tea
*Porophyllum ruderale* (Jacq.) Cass.	Papalo de venado	1	2	0	Wounds	Washing, place dried and ground on area with pain, tea
*Prosopis juliflora* (Sw.) DC.	Bejuco de Mezquite	1	0	0	Kidneys	Tea
*Prosopis laevigata* (Willd.) M.C.Johnst.	Mezquite	1	1	0	Liver	Boiled
*Psidium guajava* L.	Guayabo	1	1	45	Hangover, stomachache, diarrhea, vomiting, gastritis, colic, cough	Tea
*Psittacanthus calyculatus* (DC.) G.Don	Injerto de Huizache	1	0	2	Kidneys	Tea
*Punica granatum* L.	Granada	2	0	6	Cough, diarrhea, cancer, dysentery, thrush, cold sores, wounds	Tea, place dried and ground on area with pain, eaten, juice
*Quercus castanea* Née	Encino/Encinillo/Ahuatzitzin	1	2	0	Cough	Tea
*Randia echinocarpa* Moc. & Sessé ex DC.	Granjel/Granjel manso	1	9	3	Kidneys, wounds, cystitis	Tea, washing, eaten
*Rauvolfia tetraphylla* L.	Tlanelpolo/Tlalnenelpol	1	0	5	Postpartum, sore feet, diabetes, dengue, muscle ache, typhoid, fever	Baths, tea, alcohol
*Rhamnus humboldtiana* Willd. ex Schult.	Coyotillo	1	1	0	Toothache	Chew leaves
*Ricinus communis* L.	Higuerilla/Higuerillo	2	2	12	Indigestion, fever, stomachache	Applied to affected area and feet
*Rosa gallica* L.	Rosa de Castilla	2	0	1	Colic in babies	Baths
*Rosmarinus officinalis* L.	Romero	2	0	2	Stomachache, diabetes	Tea
*Ruellia megasphaera* Lindau	Tea negro	2	1	1	Stomachache, vomiting, diarrhea	Tea
*Ruta chalepensis* L.	Ruda	1	4	21	Bad air, indigestion, stomachache, witchcraft, headache, menstrual cramps, earache, dizziness, postpartum, anger	Baths, inhale steam, tea, rubbed over body, smeared, drops, with milk
*Sambucus canadensis* L.	Saúco	1	0	1	Cough	Tea
*Sanvitalia procumbens* Lam.	Ojo de Gallo	1	1	2	Cystitis, kidneys	Tea
*Schinus molle* L.	Pirul	2	0	2	Witchcraft, cold	Rubbed on body, tea
*Sechium edule* (Jacq.) Sw.	Chayote	1	0	1	High blood pressure	Tea
*Sedum frutescens* Rose	Siempreviva	1	0	7	Scorpion sting, eye web, irritated eyes	Drops, chewed, washing
*Sedum glaucophyllum* R.T. Clausen	Sin Vergüenza	1	0	1	Gumboil	Placed on cheeks
*Selaginella lepidophylla* (Hook. & Grev.) Spring	Doradilla/Flor de Peña	1	3	6	Kidneys, ovary problems, wounds, lungs	Tea
*Senecio salignus* DC.	Jarilla	1	0	2	Bad air, muscle ache	Rubbed over body, alcohol
*Senna hirsuta* (L.) H.S.Irwin & Barneby	Jehuite	1	1	0	Headache	Tea
*Senna skinneri* (Benth.) H.S.Irwin & Barneby	Paraca	1	6	6	Diarrhea, stomachache, indigestion, kidneys, wounds, gastritis	Tea, washing
*Senna spectabilis* (DC.) H.S.Irwin & Barneby	Candelillo	1	1	0	Ear ache	Drops
*Serjania schiedeana* Schltdl.	Palo de Tres Costillas	1	13	10	Prostate, kidneys, purify blood, bruises, lungs, burning feet, diarrhea, stomachache, inflammation,	Tea
*Simira mexicana* (Bullock) Steyerm.	Quina Roja	1	0	1	Purify blood	Tea
*Solanum lycopersicum* L.	Jitomate	2	0	3	Fever, diabetes, gums	Tea, liquified, placed on feet and belly
*Solanum marginatum* L. f.	Sacamanteca	1	0	1	Cancer	Tea
*Solanum nigrescens* M. Martens & Galeotti	Hierba Mora	1	1	0	Anger	Tea
*Solanum tuberosum* L.	Papa	2	0	1	Uric acid	Liquified
*Spinacia smelledacea* L.	Espinaca	2	0	1	Diabetes	Liquified
*Spondias mombin* L.	Ciruelo	1	0	1	Bruxism	Chewed
*Stenocereus queretaroensis* (F.A.C.Weber ex Mathes.) Buxb.	Pitahaya roja	1	0	1	Cancer	Tea
*Stevia rebaudiana* (Bertoni) Bertoni	Estevia	2	0	1	Diabetes	Tea
*Swietenia macrophylla* King	Zopilote	1	0	3	Diabetes	Tea, chewed
*Syzygium aromaticum* (L.) Merr. & L.M.Perry	Clavo	2	0	2	Toothache	Chewed
*Tagetes erecta* L.	Cempasúchil	1	1	1	Stomachache, anger	Tea
*Tagetes lucida* Cav.	Pericón	1	3	11	Stomachache, cough, postpartum, rheumatism, dengue, menstrual cramps, foot pain, wounds, anger	Tea, baths, inhaled steam
*Tamarindus indica* L.	Tamarindo	2	0	2	Constipation, parasites	Tea
*Taraxacum officinale* (Ledeb.) Schinz ex Thell.	Diente de León	2	0	2	Varicose vains, cancer	Tea
*Tecoma stans* (L.) Juss. ex Kunth	Tronadora/Histoncle	1	0	1	Diabetes	Tea
*Thymus vulgaris* L.	Tomillo	2	1	0	Stomachache	Tea
*Tilia mexicana* Schltdl.	Tila	1	0	3	Nerves	Tea
*Tournefortia hirsutissima* L.	Hierba rasposa/Tlalchichinole	1	12	26	Gastritis, diaper rash, wounds, kidneys, hand ache, stomachache, gastric ulcer, postpartum, liver, colic, cystitis	Tea, applied to affected area, infusion, washing, liquified, poultice, ground, chewed
*Tradescantia spathacea* Sw.	Maguey morado/Barquita	1	2	1	Waist ache	Tea
*Vaccinium erythrocarpum* Michx.	Arándano	2	0	1	Diabetes	Liquified
*Verbesina abscondita* Klatt	Capitaneja	1	0	2	Kidneys, muscle ache	Tea, alcohol
*Verbesina crocata* (Cav.) Less.	Capitaneja	1	1	1	Kidneys	Tea
*Vitex hemsleyi* Briq.	Querengue	1	1	0	Kidneys	Tea
*Vitex mollis* Kunth	Coyontomate	1	1	1	Cystitis, purify blood	Tea
*Vitis vinifera* L.	Pasas	2	0	1	High blood pressure	Liquified
*Waltheria indica* L.	Cuaulotillo/Tapacola/Tapaculo	1	2	12	Diarrhea, stomachache	Tea
*Zea mays L.*	Elote	1	1	4	Kidneys, cystitis, lungs	Tea
*Zornia thymifolia* Kunth	Hierba de la Vibora	1	1	1	Cold sore, snake bytes	Tea, applied to affected area

In TGO, nine key informants were interviewed—five women and four men, between the ages of 39 and 72. They held diverse occupations, from the home to traditional medicine practitioners. The majority preferred to use only medicinal plants to treat illness, since they were unfamiliar with the substances used in allopathic medicine (5), while the remaining informants preferred to combine traditional medicine with allopathic medicine.

The informants learned about the use of medicinal plants from a family member or by observing others. Several were transmitting that knowledge to their children, although many were not interested in learning, leading the informants to consider that knowledge of the use of medicinal plants was being lost in their community. In addition to the lack of interest in learning about these resources, they mentioned that these plants were disappearing and that the clinic physicians advised their patients against using medicinal plants. However, they also mentioned that one characteristic that has helped to maintain this knowledge is that the use of medicinal plants is a free alternative for those that do not have money to buy allopathic medications. They also mentioned that medicinal plants were most available in surrounding patches of native vegetation. Additionally, they commented that at the local market they could acquire many varieties of medicinal plants from other regions and even other countries. There were 127 frequently mentioned diseases or ailments treated with medicinal plants in this community, among them, kidney discomfort (32 mentions), diabetes (30), cough (26), stomachache (25), and wounds (21). They used 107 species in infusions, 28 by placing the plant directly on the painful area, and 28 in liquified preparations. Some other uses were least frequently mentioned, such as syrups and vaporizations with 2 mentions each, and using a collar made with lemons with 1 mention (Table 7).

Most of the interviewees said that they attend health clinics only when their condition does not improve with natural treatments or when they are suffering from a serious illness. In addition, medicinal plants were a source of supplementary income for these informants and their families, although the number of patients had decreased in recent years due to the increase in the availability of allopathic medicines in the community. However, thanks to the community’s confidence in their abilities, they continued to provide their services:

"…the thing is that people lose confidence in the doctors, because they say one thing, then they want to treat you for something else… I had pain from an infection I had, and the doctor checked me out and said it was my gall bladder, that I needed an operation, but I didn’t listen to him, I took some herbs I prepared and now I’m fine…but it takes time, and nowadays people don’t want to be healed, they want everything quick, and I tell them that if they want to heal, they have to take the treatment for at least a month…" Key informant from TGO (Fig. 6).

## Discussion

### The importance of tropical dry forest (TDF) in the provision of medicinal plants

The region where the study communities are located provides a considerable percentage of the medicinal plants that are sold in Mexico [68]. This indicates the importance of these resources in the culture and economy of the inhabitants of this natural reserve, as well as the contribution of the tropical dry forest (TDF) to the treatments used in, and the general persistence of, the practice of traditional medicine. In this study, we recorded a total of 217 species of medicinal plants, which correspond to 72.33% of the flora reported for the region by Maldonado-Almanza [16]. TDF is the dominant vegetation type, providing medicinal plants to the Sonora Market (*Mercado de Sonora*) in Mexico City, which is one of the most important markets for medicinal plants in Latin America [68]. Thus, the TDF is of great environmental, social, and economic importance in the conservation of these resources [36, 69–71].

This high percentage of medicinal plants (72.3%) indicates that both communities possess a fair amount of knowledge about these species due to the role they play in health and the local population’s need for viable and low-cost healthcare options. This can be explained by the fact that only 40% of the communities within the reserve have permanent public healthcare installations like those available in TGO; the inhabitants of less urbanized communities, like ELC, must invest time and money in traveling to the municipal seat to receive these services [4, 48, 72]. It is possible that these differences in access to healthcare also reflect asymmetry in knowledge of medicinal plants between the two communities.

Medicinal plants are the most important use category among the useful plants of Mexico [73] and the second most important among the group of species considered non-timber forest products (NTFPs) [ 74]. This importance is reflected by the number of ailments and treatments for which these plants are used by different local cultural groups [73]. The most represented botanical families in this study contain a large variety of useful secondary compounds in leaves, stems, bark, flowers, and fruits, in addition to being some of the most represented families in this type of vegetation [47, 73]. Species such as *Amphipterygium adstringens* (Schltdl.) Standl., *Eysenhardtia polystachya* (Ortega) Sarg., *Haematoxylum brasiletto* H. Karst., and *Crescentia alata* Kunth had the highest importance and frequency of use, which is consistent with other studies from the study area and TDF more generally [36, 53, 71, 75].

### Urbanization negatively affects the level of knowledge of locally distributed medicinal plants

In contrary to our expectation, in this study, we identified that the most urbanized community had greater overall knowledge of medicinal plants (TGO 175 spp. vs. ELC 95 spp.). This result was consistent among different criteria used to analyze the degree of knowledge of medicinal plants [11], such as the number of species mentioned, the number of native species, and the number of wild species (Table 2). This is probably related to the fact that TGO has a long history as a hub of regional distribution of medicinal plants and because it is the location of a market dating back to prehispanic times, in which merchants from all over the country gather to sell NTFP including crafts, medicinal plants, seasonal foods, utensils, beverages, etc. [76–78]. This fair generates commercial relationships and reinforces symbolic and cultural aspects that contribute to adaptation and innovation in traditional health practices as well as fomenting knowledge of medicinal plants among mestizo and semi-urban populations [9, 72, 79, 80]. Such events, Vandebroek and Balick [79] point out, allow relatively urbanized communities to maintain a large amount of knowledge of medicinal plants due to demographic and historical dynamics that often buffer the loss of this knowledge.

This dynamic of exchange of medicinal resources through the fair in TGO may also explain the increased use of introduced species and purchase (rather than gathering) of medicinal plants) in this community. As a consequence, in the more urbanized community of TGO, the number of introduced and domesticated species was a significant component of the medicinal plants known by the interviewees. In contrast, in the less urbanized community of ELC, native TDF plants and wild plants were more frequently mentioned and used. This is consistent with research by Blair that mentions that in moderately urbanized contexts, there is increased presence of useful plants, though these tend to be introduced [80]. In addition, a number of authors have proposed that in contemporary tropical pharmacopeias, people prefer introduced species to complement their therapeutic repertoir [81, 82], and in some cases, it is traditional medicine practitioners and local healers that promote and maintain introduced species within these communities [83–85]. This demonstrates the importance of valuing the knowledge and use of wild species and native domesticated species. At the same time, we found that there is greater knowledge of native and wild medicinal plants of the TDF among ELC inhabitants. This occurs because the lesser degree of urbanization results in closer proximity to wild vegetation, which favors the recognition and use of natural resources in daily life and delays the negative consequences of urbanization processes that tend to reduce human contact with their natural surroundings [11].

### Variables that influence the degree of knowledge of medicinal plants

The degree of knowledge of medicinal plants differed between the two communities (Table 2) and was affected mainly by socioeconomic variables and the age of the interviewees, the way in which they acquired medicinal plants (collection or purchase), and the number of native, introduced, wild, and domesticated plants they named (Table 4). This agrees with the assertion of Rangel de Almeida and collaborators, who explained that geographic proximity among communities is a crucial factor for their similarity in botanical knowledge [84], as occurs in our study area, where both communities are surrounded by the same type of vegetation. These differences are expressed in the type and source of the resources that are known to each group. In ELC, the people with the most knowledge of the local flora were those that work in the primary sector. In contrast, in TGO, although there was a great deal of knowledge of medicinal plants, they were most knowledgeable about exotic and domesticated plants and tended to work in the tertiary sector. These results agree with previous findings from other investigations, in which a lower level of local botanical knowledge was related to non-agricultural employment and decrease in activities related to extraction of natural resources [11].

Complementary to these differences in traditional botanical knowledge (Table 2), our findings reveal an overlap in the knowledge of medicinal plants. This can be explained by the existence of an urbanization gradient, as well as by the fact that the method of analysis compared intrinsic differences between regions within each study community (Fig. 4). These overlaps occur among most of the regions we designated, but are particularly frequent between residents of the central and peripheral regions of TGO. This may be due to family dynamics, since many people inherit plots of land from their parents which are found on the outskirts of these communities. In contrast, in ELC, homes tend to be situated on large plots of land that house the entire extended family, including children and even grandchildren. Therefore, the difference in knowledge of medicinal plants of the inhabitants of this less urbanized community could be due to the complex dynamics of migration and the establishment of people from different communities.

On the other hand, the differences in knowledge of medicinal plants could also be due to the occupation of the interviewees. While homemakers in TGO apparently mentioned a large number of medicinal plants, these were mostly purchased and introduced, which could be due to their openness to commerce, since they are the member of the family that tends to attend markets to sell farm and other products (Table 6). On the other hand, the people whose occupation was in the primary sector used more wild, collected, and domesticated native species. This is consistent with findings of Beltrán-Rodríguez and collaborators with respect to the idea that agriculture and livestock husbandry contribute to ethnobotanical knowledge, unlike those who work in commerce or service industries, which know more introduced species [69].

While it has been reported that people who work in the tertiary sector and who have higher economic income may have more knowledge of medicinal plants, it has also been observed that a considerable proportion of that knowledge is of introduced species [84, 86], which suggests that westernization and urbanization tend to homogenize local knowledge and diminish the biocultural richness of rural communities, putting at risk knowledge of medicinal plants and their natural environment [87]. The dominant culture legitimizes certain types of knowledge and practices deemed valid and desirable [88], to the point of social coercion. For example, the official health policies in Mexico often condition access to subsidies or benefits from social programs upon regular attendance of official health services, especially for inhabitants of less urbanized communities. With the understanding that culture and knowledge are flexible and dynamic, it is well known that some traditional practices are devalued by the dominant culture, which leads to transformation and erosion of experiences and knowledge of the management of the landscape and its resources [89]. For this reason, it is fundamentally important to preserve the knowledge and practices associated with the management of the natural environment, since they would disappear if there is no longer a relationship between human communities and natural elements, leading to the erosion of knowledge associated with natural resources [11, 33], the abandonment of their use [68] and their progressive loss [26].

### Threats to the continuity of use of medicinal plants in the urbanization gradient

The increasing urbanization of TGO promoted by regional migration of inhabitants from ELC to this community, as well as the constant flux of migrants between TGO and the USA could negatively affect the consumption of medicinal or other useful plant species in the region in both localities by bringing allopathic medicines or natural remedies from elsewhere, which could contribute to the destabilization of traditional identity paradigms [90]. In some studies, it has been shown that this effect can lead to cultural change, which modifies the knowledge and perception of medicinal and edible plant resources [10, 27, 79, 91].

The preference for the use of medicinal plants as a preventative method in ELC may respond to the fact that inhabitants must invest more time and money to travel to another community to receive healthcare services. Often, in order to access subsidies or benefits from the social programs of the different levels of government, the inhabitants of less urbanized communities must demonstrate that they go to official health services. This has not necessarily been reflected in an improvement in health care. Rather, it has become a mechanism by which medical personnel, some of whom have strong prejudices and are not embedded in the local culture, dismiss the remedies and preventive care derived from traditional medicine. Since physicians are viewed as an authority in many Mexican towns, in the long term, the undervaluation of traditional medicine compared to allopathic medicine becomes cemented in people’s minds. These dispositions may act as social coercion mechanisms that promote the devaluation of traditional therapies by official healthcare systems, which has negative implications for the appreciation and knowledge of medicinal plants [27, 78, 92].

In the case of ELC, the transmission of knowledge of medicinal plants is being lost, probably due to the migration of young people. Since this transmission depends on the collective memory of the communities, there is increasing tolerance of the progressive loss of knowledge of plant resources [26, 90]. It is important to mention that the key informants from ELC do not consider themselves traditional medicine practitioners, despite possessing a large body of knowledge of medicinal plants. This may imply that they do not consider it important to transmit their knowledge to others, leading to a process of colonization of the native epistemologies. This involves the dispossession and devaluing of knowledge and of the cultural foundations of indigenous, mestizo, and rural communities by the imposition of hegemonic models in multiple aspects of community life, in particular, healthcare [93–95].

The loss of this knowledge, according to interviewees, is mainly due to the speed with which allopathic medicines work and the pressure exerted by the healthcare system to disincentivize the use of medicinal plants. Both factors could result in the disuse of local resources, and therefore, disinterest in conserving them. Pérez-Nicolás and collaborators have suggested that medicinal plants cannot be used to foment forest conservation [96]. However, the case of the Flora Sanctuary *Orito Ingi Ande* in Colombia is an example that this is possible, since in 2008, the government and the indigenous community agreed to conserve the biodiversity, including many medicinal plants, and the associated traditional knowledge [97]. It is therefore important to find mechanisms that allow synergy between traditional and western healing systems. This could maintain traditional knowledge and positive valorization of natural resources, playing a positive role in the communities and the conservation of their surroundings [78, 98].

In TGO, traditional practices had an important presence in daily life and in symbolic aspects of community life. This is reflected by the knowledge possessed by its inhabitants of medicinal plants, and we therefore found a larger number of key informants that consider themselves traditional medicine practitioners. Although in both communities traditional practices are used to improve health, the cultural processes are very dynamic due to interaction with other cultures [88].

In TGO, people receive economic benefits from the use of medicinal plants, be it by collecting them, using them in traditional medicine, or using them as a cheaper alternative to allopathic medicine. This coincides with the assertion by Shackleton and collaborators that non-timber forest products are vital components for local use as well as for sale in local and regional economies [99].

The inhabitants of TGO spend less time and money to visit a health clinic and use a wider variety of forms of treatment than in ELC. We consider that having access to more healthcare options in TGO allows people to try different healing methods. In the case of ELC, it may be that since there are fewer options for treatment and lower income, in addition to a strong effect of coercion by health policies, the value of knowledge of medicinal plants decreases, with negative repercussions for their use.

Characterizing and attempting to explain complex phenomena in depth, such as the effect of urbanization on knowledge of medicinal plants, requires an interdisciplinary approach. This research highlights the value and utility of knowledge that is maintained in rural communities about their surroundings, evidencing the implications for the conservation of local flora, specifically species with medicinal uses.

## Conclusions

Knowledge of medicinal flora is diverse and dynamic, and can often be eroded by sociocultural processes like urbanization. This study shows the complexity of the phenomenon, since communities with a higher degree of urbanization can be a catalyst for the acquisition of a new set of knowledge, treatments, and forms of preparation. However, these innovations can be detrimental to the use of native flora, local knowledge systems, and their mechanisms of transmission. In this study, while the less urbanized community recognized a lower total number of medicinal plants, these were mostly native plants distributed in the surrounding vegetation. This could maintain links with and dependency on the local forest, which could stimulate conservation of important areas of tropical dry forest. Strong threats to the use of medicinal plants are evident due to complex processes, such as migration and contradictory public policy, which can erode biocultural heritage of traditional peoples.

## Data Availability

Not applicable.
